# Draft Genome Sequence of Yellow Pigmented *Jeotgalibacillus alimentarius* JY-13^T^, the First Halophile Strain of the Genus *Jeotgalibacillus*

**DOI:** 10.1128/genomeA.01224-15

**Published:** 2015-10-22

**Authors:** Amira Suriaty Yaakop, Kok-Gan Chan, Han Ming Gan, Kian Mau Goh

**Affiliations:** a Faculty of Biosciences and Medical Engineering, Universiti Teknologi Malaysia, Skudai, Johor, Malaysia; b Faculty of Science, Division of Genetics and Molecular Biology, Institute of Biological Sciences, University of Malaya, Kuala Lumpur, Malaysia; c Monash School of Science, Monash University Sunway Campus, Petaling Jaya, Selangor, Malaysia

## Abstract

*Jeotgalibacillus alimentarius* JY-13^T^ (=KCCM 80002^T^ = JCM 10872^T^) is a moderate halophile. In 2001, this was the first strain of the newly proposed *Jeotgalibacillus* genus. The draft genome of *J. alimentarius* was found to consist of 32 contigs (*N*_50_, 315,125 bp) with a total size of 3,364,745 bp. This genome information will be helpful for studies on pigmentation as well as applications for this bacterium.

## GENOME ANNOUNCEMENT

Several halophilic bacteria such as *Jeotgalibacillus alimentarius* (1), *Psychrobacter jeotgali* (2), *Methylobacterium jeotgali* (3), and *Jeotgalicoccus halotolerans* (4) have been isolated from jeotgal, a traditional Korean food (5). Some of these isolated bacteria can be used for various purposes, including antimicrobial agents (6) and fermentation starters (7). *J. alimentarius* has not been examined for its potential applications, despite the fact that this bacterium was the earliest described strain of the genus *Jeotgalibacillus*. This bacterium required NaCl to grow, and the cells are capable of tolerating up to 20% (wt/vol) salt (1). The cell exhibits a yellow pigmentation. Pigmentations in marine bacteria are often associated with the bacteria’s self-defense against harsh environmental conditions (8). Since synthetically derived pigments may be toxic, carcinogenic, or teratogenic (9), harnessing pigments from microbial resources has received increasing attention. Microbial carotenoids are pigments that are important in various applications (10). Recently, the genome sequences of *J. malaysiensis* (11), *J. soli* (12), and *J. campisalis* (13) were reported. In this study, we determined the genome sequence of *J. alimentarius* and examined the pigment formation pathway in this strain.

*J. alimentarius* genomic DNA was extracted using a DNeasy tissue kit (Qiagen, Hilden, Germany). Genome sequencing was conducted using an Illumina MiSeq sequencer (Illumina, Inc., San Diego, CA, USA). Reads were assembled using SPAdes (14) into 32 contigs with an *N*_50_ contig size of 315,125 bp. Similarity searches were conducted against several databases (CatFam, IMG-er, COG, NCBI RefSeq, and SEED). Gene prediction was conducted using Glimmer version 3.02 (Delcher et al., 1999), tRNA prediction by tRNASCAN-SE (15), and rRNA prediction by HMMER (16). The genome was 3,364,745 bp in length, with a G+C content of 43.13%. The genome was predicted to include 3,536 open reading frames, 7 rRNA genes, and 74 tRNA genes.

Generally, the formation of carotenoids in bacteria involves several steps: (1) an early reaction due to mevalonic acid to geranylgeranyl pyrophosphate; (2) phytoene formation; (3) desaturation; (4) cyclization; and (5) modifications (17). Eleven genes that are crucial for the first three steps of carotenoid formation are present in the *J. alimentarius* genome. These 11 key carotenoid-biosynthesis genes encode for an isopentenyl diphosphate isomerase, a geranylgeranyl pyrophosphate synthase, two phytoene synthases, four phytoene dehydrogenases, a fatty acid desaturase, and a glycosyltransferase. These enzymes showed about 48.5 to 98% similarities to various *Bacillus* spp., suggesting that pigmentation of *J. alimentarius* may be similar to that in most bacilli. Guo et al. (18) have performed extensive studies on phytoene dehydrogenase and concluded that this enzyme is important in the transformation of colorless carotenoid into colored compounds. The four phytoene dehydrogenase enzymes are responsible for the pigmentation of *J. alimentarius*.

### Nucleotide sequence accession numbers.

This whole-genome shotgun project has been deposited in DDBJ/ENA/GenBank under the accession number JXRQ00000000. The version described in this paper is the first version, JXRQ00000000.1.
